# TRAIL Death Receptor-4, Decoy Receptor-1 and Decoy Receptor-2 Expression on CD8^+ ^T Cells Correlate with the Disease Severity in Patients with Rheumatoid Arthritis

**DOI:** 10.1186/1471-2474-11-192

**Published:** 2010-08-27

**Authors:** Atil Bisgin, Ender Terzioglu, Cigdem Aydin, Burcak Yoldas, Veli Yazisiz, Nilufer Balci, Huseyin Bagci, Reginald M Gorczynski, Cezmi A Akdis, Salih Sanlioglu

**Affiliations:** 1 Department of Medical Genetics, Human Gene and Cell Therapy Center of Akdeniz University Hospitals and Clinics, Antalya, 07058, Turkey; 2 Division of Rheumatology and Immunology, Department of Internal Medicine, Akdeniz University Faculty of Medicine, Antalya, 07058, Turkey; 3 Department of Medical Biology and Genetics, Human Gene and Cell Therapy Center of Akdeniz University Hospitals and Clinics, Antalya, 07058, Turkey; 4 Human Gene and Cell Therapy Center of Akdeniz University Hospitals and Clinics, Antalya, 07058, Turkey; 5 Department of Physical Medicine and Rehabilitation, Akdeniz University Faculty of Medicine, Antalya, 07058, Turkey; 6 Division of Cellular & Molecular Biology, Toronto Hospital, University Health Network, Toronto, ON, Canada; 7 Swiss Institute of Allergy and Asthma Research, Davos, Switzerland

## Abstract

**Background:**

Rheumatoid Arthritis (RA) is a chronic autoimmune inflammatory disorder. Although the pathogenesis of disease is unclear, it is well known that T cells play a major role in both development and perpetuation of RA through activating macrophages and B cells. Since the lack of TNF-Related Apoptosis Inducing Ligand (TRAIL) expression resulted in defective thymocyte apoptosis leading to an autoimmune disease, we explored evidence for alterations in TRAIL/TRAIL receptor expression on peripheral T lymphocytes in the molecular mechanism of RA development.

**Methods:**

The expression of TRAIL/TRAIL receptors on T cells in 20 RA patients and 12 control individuals were analyzed using flow cytometry. The correlation of TRAIL and its receptor expression profile was compared with clinical RA parameters (RA activity scored as per DAS28) using Spearman Rho Analysis.

**Results:**

While no change was detected in the ratio of CD4^+ ^to CD8^+ ^T cells between controls and RA patient groups, upregulation of TRAIL and its receptors (both death and decoy) was detected on both CD4^+ ^and CD8^+ ^T cells in RA patients compared to control individuals. Death Receptor-4 (DR4) and the decoy receptors DcR1 and DcR2 on CD8^+ ^T cells, but not on CD4^+ ^T cells, were positively correlated with patients' DAS scores.

**Conclusions:**

Our data suggest that TRAIL/TRAIL receptor expression profiles on T cells might be important in revelation of RA pathogenesis.

## Background

Rheumatoid arthritis (RA) is a chronic autoimmune disease, which affects the synovial membrane and eventually causes irreversible destruction of tendons, cartilage, and bone [1-3]. It has long been suspected that the inflammatory lesions result from an autoimmune response to joint-specific antigens primarily involving the cells of the immune system [4-7]. Although disease commences with T cells recognizing antigen, this recognition event also drives a chronic inflammatory process involving the activation of macrophages and B cells [8,9]. The chronic inflammation is in turn perpetuated by activation of both CD4^+ ^and CD8^+ ^T cells [10].

Programmed cell death is an apoptotic mechanism by which damaged cells are removed from the body. Engagement of autoreactive T cells by self antigens within the thymus induces deletion of potentially harmful T cells. Defects in apoptosis lead to the persistence of T cells recognizing self antigens which can induce autoimmunity [11,12]. Clonally expanded T cells that have served their functional purpose are also cleared from the system through activation induced cell death (AICD) involving cell surface FasL/Fas receptor interaction [13]. Children with defective Fas-mediated T lymphocyte apoptosis exhibit a disorder known as autoimmune lymphoproliferative syndrome [14,15]. Like FasL, TNF has also been involved in AICD [16], although unlike CD95, TNF initiates and exacerbates autoimmune diseases. A third member of TNF superfamily is the TNF Related Apoptosis Inducing Ligand (TRAIL) [17]. In the immune system, TRAIL is expressed on the surface of activated T lymphocytes. Five different receptors interact with TRAIL: TRAIL Receptor-1 (TRAIL-R1/also referred to as DR4), TRAIL Receptor-2 (TRAIL-R2/DR5), TRAIL Receptor-3 (TRAIL-R3/DcR1), TRAIL Receptor-4 (TRAIL-R4/DcR2), and osteoprotegrin (OPG) [18,19]. DR4 and DR5 are the genuine death receptors inducing apoptosis whereas DcR1 and DcR2 function as decoy receptors and physiologically block apoptosis [20,21], which might thus contribute to the pathogenesis of autoimmunity [22,23]. Like CD95L, TRAIL has been reported to be a potent inhibitor of autoimmune arthritis [24]. Unlike TNF and FasL, TRAIL inhibits the activation and proliferation of lymphocytes in vivo, but does not delete them from the system.

It is apparent then that FasL, TNF and the TRAIL/TRAIL receptor system are involved in T cell activation and/or deletion [25]. Accordingly, here we demonstrate the potential usage of TRAIL and the expression profile of its receptors on peripheral T cell subsets as markers to monitor the prognosis of patients with rheumatoid arthritis.

## Methods

### Clinical Assessment of Patients with Rheumatoid Arthritis

20 RA patients and 12 age-/sex-matched control individuals were enrolled in the study conducted at the Rheumatology Clinic of Akdeniz University Hospitals. RA patients were classified according to the American Rheumatism Association 1987 revised criteria. DAS28-3 scoring (including tender joint counts, swollen joint counts and erythrocyte sedimentation rate-ESR) was used for each RA patient to assess the severity of disease. RA patients previously not treated with disease modifying anti-rheumatic drugs (anti-TNF agents) were admitted to the study. These patients had the history of receiving either non-steroidal anti-inflammatory drugs (NSAIDs) or analgesics prior to analysis.

### Collection and analysis of blood samples

Analysis of peripheral blood lymphocytes was performed by direct immunofluorescence flow cytometry using a Coulter EPICS ALTRA XL instrument. The following monoclonal antibodies (mAb) were used: Phycoerythrin (PE) anti-human DR4 (CD261, TRAIL-R1, Cat No: 12-6644-73, eBioscience Inc., San Diego, CA, USA), PE anti-human DR5 (CD262, TRAIL-R2, Cat No: 12-9908-73, eBioscience Inc., San Diego, CA, USA), PE anti-human DcR1 (CD263, TRAIL-R3, TRAILR3, LIT, TRID Cat No: 12-6238-73, eBioscience Inc., San Diego, CA, USA), PE anti-human DcR2 (CD264, TRAIL-R4, TRAILR4, TRUNDD, TNFRSF10 D Cat No: 12-6239-73, eBioscience Inc., San Diego, CA, USA), PE anti-human TRAIL (CD253 Cat No: 12-9927-73, eBioscience Inc., San Diego, CA, USA), Fluorescein isothiocyanate (FITC) anti-human CD4 (L3T4 Cat No: A07750, Beckman Coulter, Immunotech, Marseille, France), FITC anti-human CD8 (Cat No: IM0451U, Beckman Coulter, Immunotech, Marseille, France), PE Mouse IgG1 (κ Isotype Control Cat No: 12-4714-73, eBioscience Inc., San Diego, CA, USA), FITC Mouse IgG1 (κ Isotype Control Cat No: A07795, Beckman Coulter, Immunotech, Marseille, France) and FITC Mouse IgG2a (Isotype Control Cat No: 11-4724-73, eBioscience Inc., San Diego, CA, USA).

### Flow Cytometry Procedure

Reaction conditions for FC were as follows: 50 μl of each sample was diluted with 50 μl PBS solution (phosphate buffered saline; 0.01 M sodium phosphate, 0.145 M sodium chloride, Ph 7.2), and stained using 10 μl of either FITC (Fluorescein isothiocyanate) conjugated mouse monoclonal antihuman CD4 antibody or FITC conjugated mouse monoclonal antihuman CD8 antibody. PE (Phycoerythrin) conjugated mouse monoclonal antihuman TRAIL or its receptor antibodies were added subsequently to each tube. Both the activation status of T cells (CD4^+^CD25^+^) and the amount of regulatory T cells (CD4^+^CD25^+^FoxP3^+^) present were revealed using CD25 ECD (Beckman Coulter, 6607112) and APC-anti-human Foxp3 (eBioscience, 17-4776-73) antibodies. Tubes were incubated in the dark at room temperature for 20 minutes. Erythrocytes were eliminated from PBL using ammonium chloride lysing solution. After two washes with PBS, the cells were resuspended and analyzed by flow cytometry. A calibration based on lymphocyte gating was performed on EPICS Altra XL system using CD45 fluorescence and side scatter parameters prior to analysis. The peripheral blood samples were analyzed within 6 hours so that the gate could still be drawn around lymphocytes. Isotype matched antibodies were included to control for non-specific binding. All results were analyzed using Expo32 Altra software (Beckman-Coulter, Fullerton, CA).

### Ethics

Written informed consent relating to the Declaration of Helsinki was obtained from all patients. The study was approved by Akdeniz University Local Committee on Ethics.

### Statistical Analysis

The statistical package for the Social Sciences 13.0 software for Windows (SPSS Inc., Chicago, Ill) and GraphPad Prism version 5 (La Jolla, CA, USA) were used to plot the data and perform statistical analyses. In addition, SmartDraw Health Science Edition (San Diego, CA, USA) was used as a graphics software package. Normality tests were conducted using a Shapiro-Wilk method. T cell subsets (CD4^+ ^and CD8^+ ^T cells) in both groups displayed a normal distribution. Thus, a nonparametric unpaired student's T test was used to evaluate CD4^+^/CD8^+ ^T cell ratios. On the other hand, a Gaussian distribution was not observed between normal versus RA patients when analyzing TRAIL and TRAIL receptor expression profiles. For this reason, Mann-Whitney U test was used to compare CD4^+ ^versus CD8^+ ^T cell associated TRAIL marker expression in patients. All correlation analyses used Spearman's Rho tests.

## Results

### Flow Cytometric Analysis of Peripheral Blood T Lymphocytes

**Figure 1:**
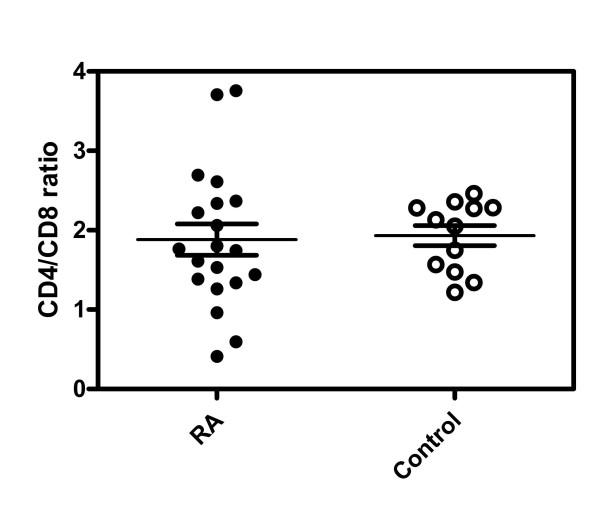
**Scatter dot plots of peripheral blood samples from 12 healthy control individuals and 20 RA patients showing the ratio of T cell subsets (CD4^+ ^and CD8^+^) by flow cytometry**.

Given the heterogeneous nature of rheumatoid arthritis, and evidence for variation in the ratio of CD4^+^/CD8^+ ^T cells changes in autoimmune diseases such as Systemic Lupus Erythematosus (SLE) [26-28], we first characterized the composition of peripheral blood T cell subsets in RA patients using flow cytometry. Nonparametric unpaired student's T test was used to detect possible differences in the two T cell subsets between normal versus RA patients. As shown in Figure 1, no difference was detected in the ratio of CD4^+ ^(p = 0.63) to CD8^+ ^(p = 0.22) T cells between the two groups. We conclude that, unlike Systemic Lupus Erythematosus, disease status does not alter the ratio of CD4^+ ^to CD8^+ ^T cell subsets in RA.

### Membrane bound TRAIL and its receptor expression profile on peripheral CD4^+ ^T lymphocytes in RA patients versus control individuals

**Figure 2:**
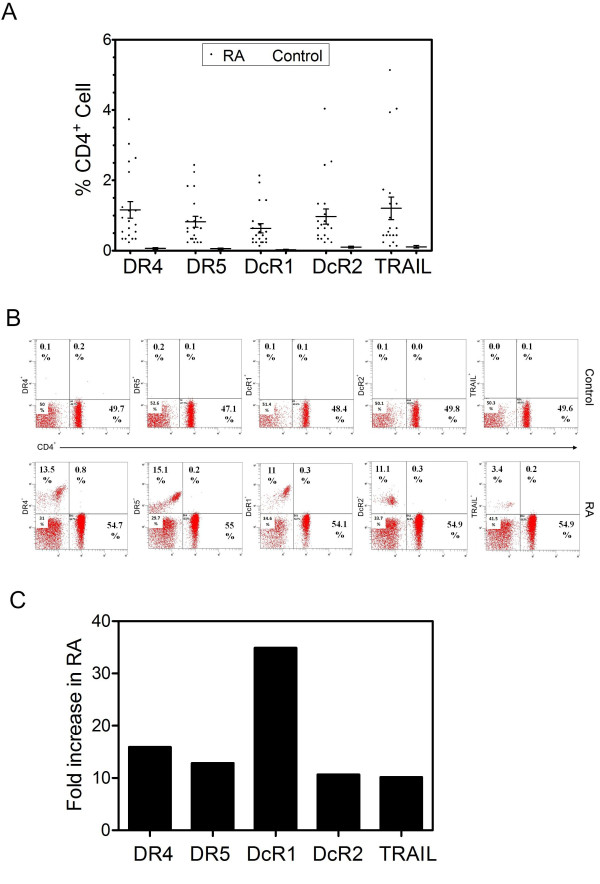
**TRAIL and its receptor (both death and decoy) expression profile on CD4^+ ^T cells**. A scattered dot plot profile of TRAIL and its receptors is provided in **Panel A**. Solid circles indicate RA patients while dots represent control individuals. Error bars display mean (±) SEM. Representative flow cytometric analyses of a control individual (upper panels) and an RA patient (lower panels) are shown in **Panel B**, while **Panel C **shows the relative increase in TRAIL and its receptors on CD4^+ ^T cells of RA patients compared to controls.

During the development of RA, CD4^+ ^T cells initiate and regulate several cell-mediated immune processes that cause synovial inflammation and joint destruction in response to activation by antigen presentation. We next examined the CD4*^+ ^*T lymphocyte associated cell-surface expression profile of TRAIL and its receptors isolated from either healthy volunteers or RA patients using flow cytometry. The Mann-Whitney U test was used to compare the two groups. As shown in Figure 2A, CD4*^+ ^*T cells obtained from RA patients exhibited higher levels of expression of TRAIL and its death/decoy receptors compared to cells from control individuals (p < 0.05). Representative flow cytometric analyses of CD4*^+ ^*T cells isolated from a healthy individual and an RA patient are shown in Figure 2B, while Figure 2C indicates the relative increase in marker expression levels. These data show a 35-fold increase in DcR1 expression with a 10-15-fold increase in the other markers.

Using a Spearman Rho correlation test to evaluate correlations between expression of TRAIL and TRAIL receptors on CD4^+ ^T cells in RA patients, we found increased DR4 expression was correlated with an increase in both DR5 and TRAIL (Table 1). In addition, DcR1 and DcR2 expression showed strong correlation (p < 0.01).

**Table 1:** Spearman rho correlation analysis of TRAIL and its receptors present on CD4^+ ^T cells isolated from RA patients.

RA-CD4 Spearman's Rho		DR4	DR5	DcR1	DcR2	TRAIL
DR4	correlation Coefficient	1.000	.620(**)	.116	.255	.764(**)
	Sig. (2-tailed)	.	.004	.626	.277	.000
	N	20	20	20	20	20

DR5	correlation Coefficient	.620(**)	1.000	.470(**)	.416	.374
	Sig. (2-tailed)	.004	.	.037	.068	.104
	N	20	20	20	20	20

DcR1	correlation Coefficient	.116	.470(**)	1.000	.639(**)	.086
	Sig. (2-tailed)	.626	.037	.	.002	.718
	N	20	20	20	20	20

DcR2	correlation Coefficient	.255	.416	.639(**)	1.000	.414
	Sig. (2-tailed)	.277	.068	.002	.	.070
	N	20	20	20	20	20

TRAIL	correlation Coefficient	.764(**)	.374	.086	.414	1.000
	Sig. (2-tailed)	.000	.104	.718	.070	.
	N	20	20	20	20	20

** Correlation is significant at the 0.01 level (2-tailed).

* Correlation is significant at the 0.05 level (2-tailed).

### CD8^+ ^T cell associated TRAIL and its receptor expression profile in RA

**Figure 3:**
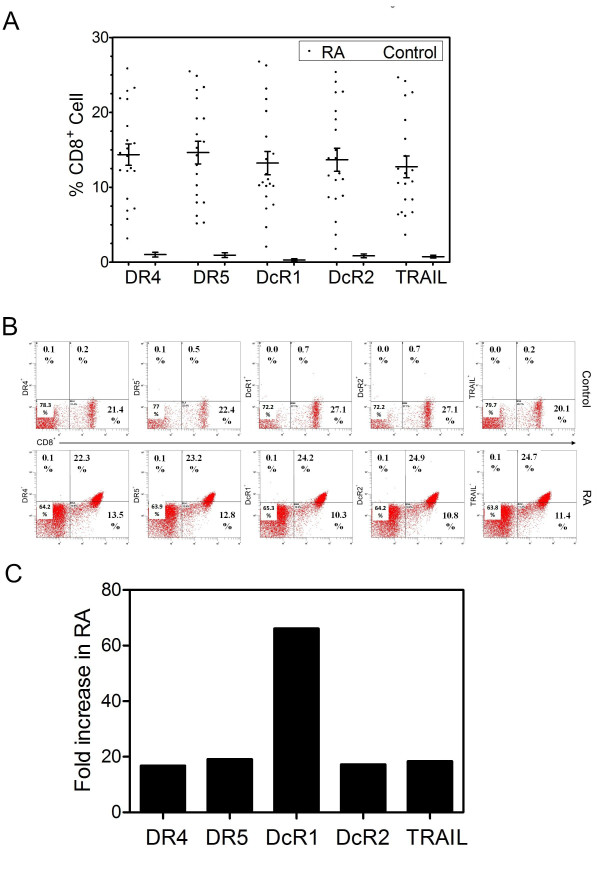
**Panel A: CD8^+ ^T cell associated TRAIL and its receptor expression profile**. TRAIL and its receptor expression profile of CD8^+ ^T cells gathered from 20 RA patients and 12 healthy controls are shown as a scatter dot plot. Error bars represent mean (±) SEM. **Panel B **shows representative FC for TRAIL and its receptor expression on CD8^+ ^T cells (upper panels show healthy control, lower panels are obtained from an RA patient). **Panel C **shows the relative increase in TRAIL and its receptors on CD8^+ ^T cells on RA cells (vs control).

We next investigated the expression of TRAIL and its receptors on CD8*^+ ^*T cells of RA patients and compared with healthy controls, similar to CD4*^+ ^*T cells, we found significant differences between the two groups as shown in Figure 3A (p < 0.05). Representative FC data from a patient and control are shown in Figure 3B, with Figure 3C indicating the relative increase in CD8^+ ^T cell associated TRAIL and its receptor expression. In this case increased DcR1 expression (about 66 fold) was the most marked. A correlation between CD8^+ ^T cell associated TRAIL and receptor expression was investigated using Spearmen Rho Correlation analysis. As shown in Table 2, a correlation among the death and the decoy receptors as well as with TRAIL death ligand expression was observed.

**Table 2:** Spearman rho correlation analysis of CD8^+ ^T cell associated TRAIL and its receptors obtained from RA patients.

RA-CD8 Spearman's Rho		DR4	DR5	DcR1	DcR2	TRAIL
DR4	correlation Coefficient	1.000	.868(**)	.948(**)	.740(**)	.744(**)
	Sig. (2-tailed)	.	.004	.000	.000	.000
	N	20	20	20	20	20

DR5	correlation Coefficient	.868(**)	1.000	.784(**)	.738(**)	.632(**)
	Sig. (2-tailed)	.000	.	.000	.000	.003
	N	20	20	20	20	20

DcR1	correlation Coefficient	.948(**)	.784(**)	1.000	.773(**)	.689(**)
	Sig. (2-tailed)	.000	.000	.	.000	.001
	N	20	20	20	20	20

DcR2	correlation Coefficient	.740(**)	.738(**)	.773(**)	1.000	.525(*)
	Sig. (2-tailed)	.000	.000	.000	.	.017
	N	20	20	20	20	20

TRAIL	correlation Coefficient	.744(**)	.632(**)	.689(**)	.525(*)	1.000
	Sig. (2-tailed)	.000	.003	.001	.017	.
	N	20	20	20	20	20

** Correlation is significant at the 0.01 level (2-tailed).

* Correlation is significant at the 0.05 level (2-tailed).

A non-parametric Mann-Whitney U test was administered to investigate statistical differences in expression levels between CD4^+ ^and CD8^+ ^T cell associated TRAIL and its receptors in patients with RA. Our results indicated that expression levels of TRAIL and its receptors were higher on CD8^+ ^T cells (Figure 3A) than on CD4^+ ^T cells (Figure 2A).

### CD8^+ ^T cell associated DR4, DcR1 and DcR2 expression levels correlated with DAS28 scores in RA patients

**Figure 4:**
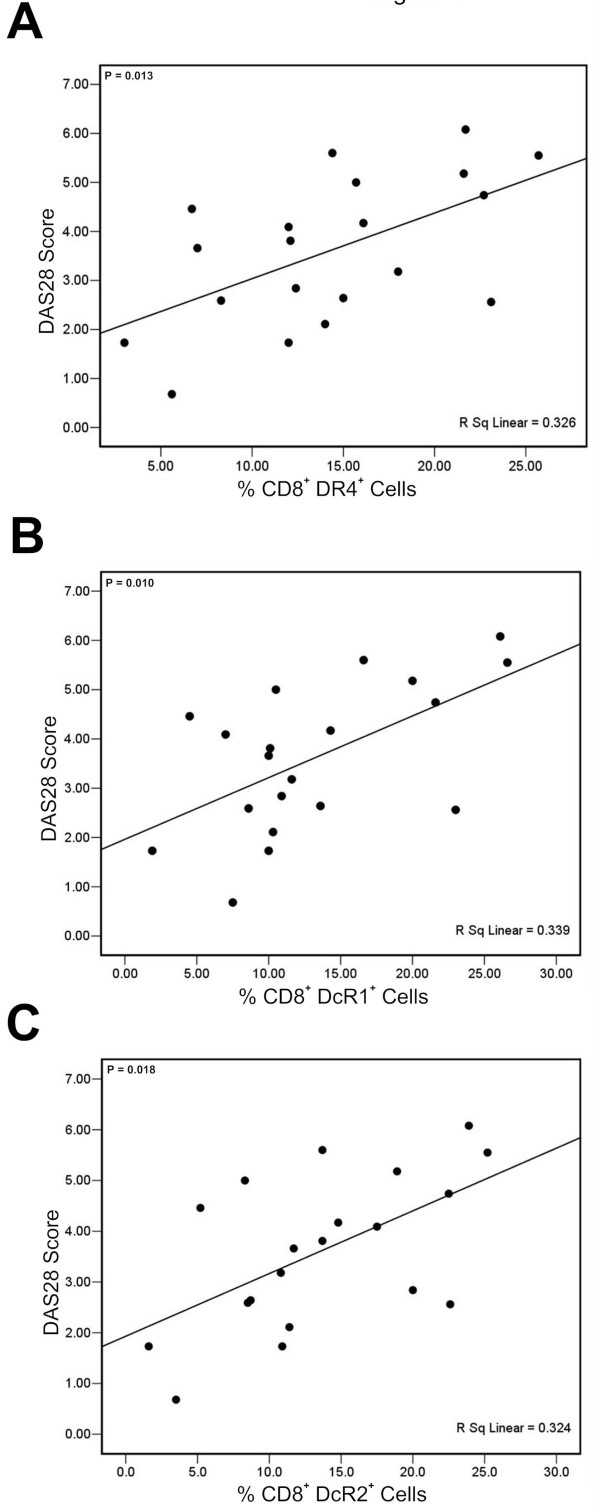
**Correlation between DR4^+ ^(Panel A), DcR1^+ ^(Panel B) and DcR2^+ ^(Panel C) expression levels and RA disease activity (DAS28 Score)-Spearman Rho correlation analysis**.

Finally we investigated evidence for a correlation between TRAIL and its receptor expression profile and the severity of disease in RA patients. Interestingly only CD8^+ ^T cell associated (Figure 4) but not CD4^+ ^T cell associated (data not shown) DR4, DcR1 and DcR2 expression levels correlated with DAS28 scores in RA patients.

### T cell activation status of newly diagnosed RA patients

CD25 is one commonly used marker for recently activated T cells [29]. In order to document the activation status of T cells in RA patients; another set of flow cytometry assay was conducted. As shown in Table 3, the percentage of CD4^+^CD25^+ ^T cells was statistically higher in RA patients compared to control individuals. However, as shown before both activated and regulatory T cells (T_reg_) can express CD25 marker on the cell surface [30]. To distinguish these two, FoxP3 staining was employed as described in Materials and Methods. No difference was noted in the amount of T_reg _(CD4^+^CD25^+ ^FoxP3^+^) between RA and control patients (Table 3).

**Table 3:** T cell activation marker profile and T_reg _status in RA versus healthy control patients.

Cell Subsets	RA (% cell ± SD)	Control (% cell ± SD)	p value
CD4^+ ^CD25^+^	4,9 ± 2,9	3,1 ± 2,0	0,001

CD4^+ ^CD25^+ ^FoxP3^+^	0,8 ± 1,9	0,6 ± 0,4	0,259

## Discussion

It has previously been reported that negative selection of T cells in the thymus is controlled by TRAIL [25]. For example, mice deficient in TRAIL had a severe defect in thymic deletion of T cells and were hypersensitive to collagen-induced arthritis [31]. Evidence for functional consequences of TRAIL over-expression in autoimmune diseases including rheumatoid arthritis has emerged from gene therapy studies [32]. Although TRAIL is not cytotoxic to normal cells [33], hyperplastic human RA synovial fibroblasts in culture and RA-activated rabbit synovial tissue *in vivo *were sensitive to adenoviral delivery of human TRAIL [34]. The effect of TRAIL expression on blood lymphocytes was reported to be different (killing versus suppression) than that observed with RA synovial cells. These studies suggested that TRAIL can inhibit the activation and proliferation of lymphocytes *in vivo*, but does not necessarily delete them from the system [35].

The TRAIL/TRAIL receptor system has recently been implicated as a disease activation marker in cancer. High DcR2 expression levels in patients with prostate cancer (PCa) indicate a poorer prognosis, with this marker strongly correlated with high Gleason Scores, Prostate Specific Antigen (PSA) recurrence and decreased survival in PCa patients [36]. In patients with invasive ductal carcinoma, however, high levels of DR4 expression are positively correlated with tumor grade and with poor prognosis [37]. Two recent studies have linked high levels of endogenous TRAIL expression to increased cell death in human pancreatic tissue, supporting the idea that TRAIL might be implicated in the development of autoimmune diseases such as Type 1 Diabetes [38,39]. Despite this information, there is no information concerning the profile of the TRAIL/TRAIL receptor system in the development of RA.

We analyzed peripheral blood lymphocytes of 20 RA patients and 12 control individuals by flow cytometry to detect their TRAIL/TRAIL receptor profile. T cell lymphocytes of healthy control individuals expressed low levels of TRAIL and TRAIL receptors on the cell surface. While this is in accordance with a study conducted by Lu et al. [40], Hasegawa et al. have shown that only DcR2 expression but no other TRAIL receptor expression was detectable on CD8+ T cells [41]. Differences between these studies including ours could be attributed to differences in monoclonal antibodies used. Nevertheless, both CD4^+ ^and CD8^+ ^T lymphocytes of RA patients displayed higher levels of TRAIL and its receptors on the cell surface compared to healthy control individuals. Since PCa patients can be separated from patients with benign prostate hyperplasia by the amount of TRAIL/TRAIL receptor present in prostate tissue [42] our observations suggest that it might be useful to monitor TRAIL/TRAIL receptor expression in peripheral blood lymphocytes in RA patients at diagnosis and during the course of their disease. The most dramatic change in the amount of TRAIL/TRAIL receptor present on peripheral blood lymphocytes of RA patients was the increased DcR1 expression seen in both CD4^+ ^and CD8^+ ^T cells. Recently, elevated expression of DcR1 was detected in antigen specific T cells of patients with multiple sclerosis (MS) [22]. These antigen specific T cell clones were also resistant to TRAIL mediated apoptosis. One interpretation of these data is that antigen specific T cell clones increase their survival following DcR1 upregulation.

Based on our flow cytometry analysis, RA patients displayed higher levels of activated T cells compared to healthy controls. This finding is in accordance with previous studies demonstrating the presence of higher levels of CD4^+^CD25^+ ^peripheral blood lymphocytes in RA patients compared to healthy individuals [43,44]. However we did not observe any change in the level of CD4^+^CD25^+ ^FoxP3^+ ^T cells between RA and control groups. In accordance with previous studies, no relationship was found between disease activity and CD4^+^CD25^+ ^or CD4^+^CD25^+ ^FoxP3^+ ^T cells in RA patients [45]. Whether any subtle increase in TRAIL or TRAIL receptor expression observed on CD4+ T cells in RA patients is simply a consequence of T cell activation remains to be clarified.

## Conclusions

Clinical evaluation of RA patients involves RA activity scoring (DAS28). Intriguingly, only the CD8^+ ^T cell associated DR4, DcR1 and DcR2 expression levels correlated with DAS28 scores in patients with RA, implying that altered TRAIL receptor profiles on CD8^+ ^T cell subsets rather than on CD4^+ ^T cells is more important in terms of disease severity.

## Competing interests

The authors declare that they have no competing interests.

## Authors' contributions

AB, CA and BY carried out all the assays, ET, NB and VY provided clinical data, RMG and CAA were responsible with flow cytometry procedures, HB acted as scientific consultant, SS coordinated and supervised the study. All the authors read and approved the final manuscript.

## Pre-publication history

The pre-publication history for this paper can be accessed here:

http://www.biomedcentral.com/1471-2474/11/192/prepub

## References

[B1] Koch AE Review: angiogenesis: implications for rheumatoid arthritis Arthritis Rheum 1998 41 6 951 962 10.1002/1529-0131(199806)41:6<951::AID-ART2>3.0.CO;2-D 9627005

[B2] Walsh DA Angiogenesis and arthritis Rheumatology (Oxford) 1999 38 2 103 112 10.1093/rheumatology/38.2.103 10342621

[B3] Yin G Liu W An P Li P Ding I Planelles V Schwarz EM Min W Endostatin gene transfer inhibits joint angiogenesis and pannus formation in inflammatory arthritis Mol Ther 2002 5 5 Pt 1 547 554 10.1006/mthe.2002.0590 11991745

[B4] Panayi GS T-cell-dependent pathways in rheumatoid arthritis Curr Opin Rheumatol 1997 9 3 236 240 10.1097/00002281-199705000-00010 9204259

[B5] Weyand CM Goronzy JJ Pathogenesis of rheumatoid arthritis Med Clin North Am 1997 81 1 29 55 10.1016/S0025-7125(05)70504-6 9012754

[B6] Nepom GT Major histocompatibility complex-directed susceptibility to rheumatoid arthritis Adv Immunol 1998 68 315 332 full_text 9505093 10.1016/s0065-2776(08)60563-5

[B7] Koetz K Bryl E Spickschen K O'Fallon WM Goronzy JJ Weyand CM T cell homeostasis in patients with rheumatoid arthritis Proc Natl Acad Sci USA 2000 97 16 9203 9208 10.1073/pnas.97.16.9203 10922071 PMC16846

[B8] Weyand CM Goronzy JJ T-cell-targeted therapies in rheumatoid arthritis Nat Clin Pract Rheumatol 2006 2 4 201 210 10.1038/ncprheum0142 16932686

[B9] Smolen JS What is the place of recently approved T cell-targeted and B cell-targeted therapies in the treatment of rheumatoid arthritis? Lessons from global clinical trials J Rheumatol Suppl 2007 79 15 20 17611974

[B10] Lefkowitz DL Lefkowitz SS Macrophage-neutrophil interaction: a paradigm for chronic inflammation revisited Immunol Cell Biol 2001 79 5 502 506 10.1046/j.1440-1711.2001.01020.x 11564158

[B11] Stuart L Hughes J Apoptosis and autoimmunity Nephrol Dial Transplant 2002 17 5 697 700 10.1093/ndt/17.5.697 11981048

[B12] Marleau AM Sarvetnick N T cell homeostasis in tolerance and immunity J Leukoc Biol 2005 78 3 575 584 10.1189/jlb.0105050 15894586

[B13] Singer GG Carrera AC Marshak-Rothstein A Martinez C Abbas AK Apoptosis, Fas and systemic autoimmunity: the MRL-lpr/lpr model Curr Opin Immunol 1994 6 6 913 920 10.1016/0952-7915(94)90013-2 7536012

[B14] Fisher GH Rosenberg FJ Straus SE Dale JK Middleton LA Lin AY Strober W Lenardo MJ Puck JM Dominant interfering Fas gene mutations impair apoptosis in a human autoimmune lymphoproliferative syndrome Cell 1995 81 6 935 946 10.1016/0092-8674(95)90013-6 7540117

[B15] Drappa J Vaishnaw AK Sullivan KE Chu JL Elkon KB Fas gene mutations in the Canale-Smith syndrome, an inherited lymphoproliferative disorder associated with autoimmunity N Engl J Med 1996 335 22 1643 1649 10.1056/NEJM199611283352204 8929361

[B16] Lawrence CP Chow SC FADD deficiency sensitises Jurkat T cells to TNF-alpha-dependent necrosis during activation-induced cell death FEBS Lett 2005 579 28 6465 6472 10.1016/j.febslet.2005.10.041 16289096

[B17] Sanlioglu AD Koksal T Baykara M Luleci G Karacay B Sanlioglu S Current progress in adenovirus mediated gene therapy for patients with prostate carcinoma Gene Ther Mol Biol 2003 7 113 133

[B18] Griffith TS Lynch DH TRAIL: a molecule with multiple receptors and control mechanisms Curr Opin Immunol 1998 10 5 559 563 10.1016/S0952-7915(98)80224-0 9794836

[B19] Sanlioglu AD Koksal IT Karacay B Baykara M Luleci G Sanlioglu S Adenovirus-mediated IKKbetaKA expression sensitizes prostate carcinoma cells to TRAIL-induced apoptosis Cancer Gene Ther 2006 13 1 21 31 10.1038/sj.cgt.7700877 16052230

[B20] Griffith TS Rauch CT Smolak PJ Waugh JY Boiani N Lynch DH Smith CA Goodwin RG Kubin MZ Functional analysis of TRAIL receptors using monoclonal antibodies J Immunol 1999 162 5 2597 2605 10072501

[B21] Sanlioglu AD Karacay B Koksal IT Griffith TS Sanlioglu S DcR2 (TRAIL-R4) siRNA and adenovirus delivery of TRAIL (Ad5hTRAIL) break down in vitro tumorigenic potential of prostate carcinoma cells Cancer Gene Ther 2007 14 12 976 984 10.1038/sj.cgt.7701087 17853923

[B22] Wendling U Walczak H Dorr J Jaboci C Weller M Krammer PH Zipp F Expression of TRAIL receptors in human autoreactive and foreign antigen-specific T cells Cell Death Differ 2000 7 7 637 644 10.1038/sj.cdd.4400692 10889508

[B23] Dirice E Sanlioglu AD Kahraman S Ozturk S Balci MK Omer A Griffith TS Sanlioglu S Adenovirus-mediated TRAIL gene (Ad5hTRAIL) delivery into pancreatic islets prolongs normoglycemia in streptozotocin-induced diabetic rats Hum Gene Ther 2009 20 10 1177 1189 10.1089/hum.2009.039 19572802

[B24] Song K Chen Y Goke R Wilmen A Seidel C Goke A Hilliard B Chen Y Tumor necrosis factor-related apoptosis-inducing ligand (TRAIL) is an inhibitor of autoimmune inflammation and cell cycle progression J Exp Med 2000 191 7 1095 1104 10.1084/jem.191.7.1095 10748228 PMC2193179

[B25] Tsokos GC Tsokos M The TRAIL to arthritis J Clin Invest 2003 112 9 1315 1317 14597758 10.1172/JCI20297 PMC228491

[B26] Maeda N Sekigawa I Iida N Matsumoto M Hashimoto H Hirose S Relationship between CD4+/CD8+ T cell ratio and T cell activation in systemic lupus erythematosus Scand J Rheumatol 1999 28 3 166 170 10.1080/03009749950154248 10380839

[B27] Matsushita M Hayashi T Ando S Sekigawa I Iida N Hashimoto H Hirose S Changes of CD4/CD8 ratio and interleukin-16 in systemic lupus erythematosus Clin Rheumatol 2000 19 4 270 274 10.1007/PL00011171 10941806

[B28] Wang H Xu J Ji X Yang X Sun K Liu X Shen Y The abnormal apoptosis of T cell subsets and possible involvement of IL-10 in systemic lupus erythematosus Cell Immunol 2005 235 2 117 121 10.1016/j.cellimm.2005.08.031 16226734

[B29] Cao D Malmstrom V Baecher-Allan C Hafler D Klareskog L Trollmo C Isolation and functional characterization of regulatory CD25brightCD4+ T cells from the target organ of patients with rheumatoid arthritis Eur J Immunol 2003 33 1 215 223 10.1002/immu.200390024 12594850

[B30] Baecher-Allan C Brown JA Freeman GJ Hafler DA CD4+CD25high regulatory cells in human peripheral blood J Immunol 2001 167 3 1245 1253 11466340 10.4049/jimmunol.167.3.1245

[B31] Lamhamedi-Cherradi SE Zheng SJ Maguschak KA Peschon J Chen YH Defective thymocyte apoptosis and accelerated autoimmune diseases in TRAIL-/- mice Nat Immunol 2003 4 3 255 260 10.1038/ni894 12577054

[B32] Terzioglu E Bisgin A Sanlioglu AD Ulker M Yazisiz V Tuzuner S Sanlioglu S Concurrent gene therapy strategies effectively destroy synoviocytes of patients with rheumatoid arthritis Rheumatology (Oxford) 2007 46 5 783 789 10.1093/rheumatology/kel448 17309888

[B33] Aydin C Sanlioglu AD Karacay B Ozbilim G Dertsiz L Ozbudak O Akdis CA Sanlioglu S Decoy receptor-2 small interfering RNA (siRNA) strategy employing three different siRNA constructs in combination defeats adenovirus-transferred tumor necrosis factor-related apoptosis-inducing ligand resistance in lung cancer cells Hum Gene Ther 2007 18 1 39 50 10.1089/hum.2006.111 17187448

[B34] Yao Q Wang S Gambotto A Glorioso JC Evans CH Robbins PD Ghivizzani SC Oligino TJ Intra-articular adenoviral-mediated gene transfer of trail induces apoptosis of arthritic rabbit synovium Gene Ther 2003 10 12 1055 1060 10.1038/sj.gt.3301881 12776164

[B35] Liu Z Xu X Hsu HC Tousson A Yang PA Wu Q Liu C Yu S Zhang HG Mountz JD CII-DC-AdTRAIL cell gene therapy inhibits infiltration of CII-reactive T cells and CII-induced arthritis J Clin Invest 2003 112 9 1332 1341 14597760 10.1172/JCI19209 PMC228459

[B36] Koksal IT Sanlioglu AD Karacay B Griffith TS Sanlioglu S Tumor necrosis factor-related apoptosis inducing ligand-R4 decoy receptor expression is correlated with high Gleason scores, prostate-specific antigen recurrence, and decreased survival in patients with prostate carcinoma Urol Oncol 2008 26 2 158 165 18312935 10.1016/j.urolonc.2007.01.022

[B37] Sanlioglu AD Korcum AF Pestereli E Erdogan G Karaveli S Savas B Griffith TS Sanlioglu S TRAIL death receptor-4 expression positively correlates with the tumor grade in breast cancer patients with invasive ductal carcinoma Int J Radiat Oncol Biol Phys 2007 69 3 716 723 17512128 10.1016/j.ijrobp.2007.03.057

[B38] Sanlioglu AD Dirice E Elpek O Korcum AF Balci MK Omer A Griffith TS Sanlioglu S High levels of endogenous tumor necrosis factor-related apoptosis-inducing ligand expression correlate with increased cell death in human pancreas Pancreas 2008 36 4 385 393 10.1097/MPA.0b013e318158a4e5 18437085

[B39] Sanlioglu AD Dirice E Elpek O Korcum AF Ozdogan M Suleymanlar I Balci MK Griffith TS Sanlioglu S High TRAIL Death Receptor 4 and Decoy Receptor 2 Expression Correlates With Significant Cell Death in Pancreatic Ductal Adenocarcinoma Patients Pancreas 2009 38 2 154 160 10.1097/MPA.0b013e31818db9e3 18981952

[B40] Lum JJ Pilon AA Sanchez-Dardon J Phenix BN Kim JE Mihowich J Jamison K Hawley-Foss N Lynch DH Badley AD Induction of cell death in human immunodeficiency virus-infected macrophages and resting memory CD4 T cells by TRAIL/Apo2l J Virol 2001 75 22 11128 11136 10.1128/JVI.75.22.11128-11136.2001 11602752 PMC114692

[B41] Hasegawa H Yamada Y Harasawa H Tsuji T Murata K Sugahara K Tsuruda K Masuda M Takasu N Kamihira S Restricted expression of tumor necrosis factor-related apoptosis-inducing ligand receptor 4 in human peripheral blood lymphocytes Cell Immunol 2004 231 1-2 1 7 10.1016/j.cellimm.2004.11.001 15919363

[B42] Sanlioglu AD Koksal IT Ciftcioglu A Baykara M Luleci G Sanlioglu S Differential expression of TRAIL and its receptors in benign and malignant prostate tissues J Urol 2007 177 1 359 364 10.1016/j.juro.2006.08.087 17162091

[B43] Ehrenstein MR Evans JG Singh A Moore S Warnes G Isenberg DA Mauri C Compromised function of regulatory T cells in rheumatoid arthritis and reversal by anti-TNFalpha therapy J Exp Med 2004 200 3 277 285 10.1084/jem.20040165 15280421 PMC2211983

[B44] van Amelsfort JM Jacobs KM Bijlsma JW Lafeber FP Taams LS CD4(+)CD25(+) regulatory T cells in rheumatoid arthritis: differences in the presence, phenotype, and function between peripheral blood and synovial fluid Arthritis Rheum 2004 50 9 2775 2785 10.1002/art.20499 15457445

[B45] Han GM O'Neil-Andersen NJ Zurier RB Lawrence DA CD4+CD25high T cell numbers are enriched in the peripheral blood of patients with rheumatoid arthritis Cell Immunol 2008 253 1-2 92 101 10.1016/j.cellimm.2008.05.007 18649874 PMC2585376

